# An empirical study of modified frontolateral partial laryngectomy without tracheotomy

**DOI:** 10.3892/etm.2012.838

**Published:** 2012-11-28

**Authors:** HONGMING XU, PIN DONG, ZHENFENG SUN, JIN XIE

**Affiliations:** 1 Department of Otolaryngology, Head and Neck Surgery, Shanghai Jiao Tong University Medical College, Affiliated Shanghai First People’s Hospital; Shanghai, P.R. China; 2 Department of Otolaryngology, Shanghai Jiao Tong University Medical College, Affiliated Shanghai Children’s Hospital, Shanghai, P.R. China

**Keywords:** animal experiment, carcinoma, laryngectomy, tracheotomy

## Abstract

The aim of this study was to validate the feasibility of modified frontolateral partial laryngectomy without tracheotomy using animal experiments. The glottic area before and after surgery of 6 excised canine larynges and 10 canine larynges *in vivo* were compared to observe whether the glottic area following modified frontolateral partial laryngectomy without tracheotomy is adequate for breathing. Significant differences were observed between the glottic areas of the excised larynges in the initial state and following modified frontolateral partial laryngectomy with the cartilage closed. However, no significant differences were observed between the glottic areas of the excised larynx in the initial state and following modified frontolateral partial laryngectomy with the cartilage open. The glottic area of the larynges *in vivo* in the initial state and following right chordectomy via laryngofissure were not observed to be significantly different. Furthermore, no significant differences were observed between the glottic areas of the larynges *in vivo* in the initial state and following modified frontolateral partial laryngectomy without tracheotomy. In conclusion, modified frontolateral partial laryngectomy without tracheotomy is a feasible and efficacious means of eradicating early and selected invasive carcinomas of the larynx, which is supported by animal experiments.

## Introduction

Frontolateral partial laryngectomy and laryngofissure for cordectomy are commonly used conservative approaches in the treatment of early laryngeal cancer (1–3). In these procedures, tracheotomy is a standard intervention used to alleviate dyspnea caused by postoperative edema and the consequent shrinking of the laryngeal cavity. The goals of oncological surgery of the larynx are to remove the local disease and conserve the physiological functioning of the larynx, while preserving quality of life. Therefore, a number of procedures have been proposed to minimize the extent of postoperative morbidity. Rebeiz *et al*(4) reported the successful completion of a combined endoscopic and open approach without tracheotomy for T1 and T2 glottic and supraglottic cancers in a series of 5 patients. In the case of small primary laryngeal tumors, traditional partial laryngectomy is often considered to be too aggressive due to the associated complications. We have previously described the development of a novel approach, modified frontolateral partial laryngectomy without tracheotomy, and reviewed the results in 65 patients who underwent this new procedure (5). In the present study, animal experiments were performed in order to objectively evaluate the airway provided by modified frontolateral partial laryngectomy without tracheotomy compared with other commonly used procedures.

## Materials and methods

### Animals

The present study was approved by the Animal Research Ethics Committee of Shanghai Jiao Tong University Medical College (Affiliated Shanghai First People’s Hospital, Shanghai, China) and was conducted in strict compliance with its requirements and decisions. Male and female mongrel dogs of various ages, weighing 9–15 kg were used for the experiment. A total of 16 dogs were divided into 2 groups denoted *ex vivo* and *in vivo*. The *ex vivo* group contained 6 dogs and the *in vivo* group contained 10 dogs.

### Experimental procedures of the ex vivo group

The dogs were sacrificed and their larynges were excised for anatomical study and placed in appropriate individual plastic vessels. The supraglottic tissue was removed to expose the true vocal folds. Stepwise procedures were performed and, after each step, the vocal folds were photographed from a superior perspective using a grid placed over the vocal folds to measure the glottal area.

Photographic images of the glottis were captured prior to performing any procedures on the larynx. The thyroid laminae were then incised vertically 2–3 mm posterior to the anterior commissure and removed. In all larynges, ∼20% of the laryngeal cavity was excised. The incisal margins of the vocal folds were sutured to the ipsilateral thyroid perichondrium and the glottis was then photographed. Finally, the incisal edges of the thyroid laminae were sutured together to reconstruct the anterior commissure. The glottis was then photographed for the third time.

### Experimental procedures of the in vivo group

The laryngeal cavity was isolated and adequately exposed. The epiglottis was captured and pulled forward and upward after the mucosa of the epiglottic vallecula was cut open. The mucosa of the lateral pharyngeal walls was cut to pull the whole larynx out. Photographic images of the glottis were then captured at the maximum phase to measure the maximum phase area of the untreated glottis. Right cordectomy was performed via laryngofissure and the incisal margins of the thyroid cartilage were sutured together; the glottis was then photographed again at maximum phase. Subsequently, modified frontolateral partial laryngectomy was performed according to the procedure described previously (5,6) and the maximum phase of the glottis was photographed for the third time.

### Data processing

Images of the *ex vivo* group were processed using AutoCAD2004 image software and the following data were obtained: i) area of the untreated glottis of the *ex vivo* larynx; ii) area of the glottis of the *ex vivo* larynx with the incisal margins of the thyroid cartilage sutured following frontolateral partial laryngectomy; and iii) area of the glottis of the *ex vivo* larynx following frontolateral partial laryngectomy with the incisal margins of the anterior commissure sutured to the homolateral perichondrium of the thyroid cartilage.

Images of the *in vivo* group were processed using AutoCAD2004 image software and the following data were obtained: i) the maximum phase area of the untreated glottis of the *in vivo* larynx; ii) the maximum phase area of the glottis of the *in vivo* larynx following right cordectomy via laryngofissure; and iii) the maximum phase area of the glottis of the *in vivo* larynx following modified frontolateral partial laryngectomy.

### Statistical analysis

The data of *ex vivo* experiments were compared by Student’s t-test, since the data were normally distributed. The data of the *in vivo* group was compared using ANOVA and Newman Keuls test.

## Results

### Experimental results of the ex vivo group

The *ex vivo* glottises were stationary and the areas were relatively constant without the effect of respiration and thus simple to measure. The distance and angle of the scales may greatly affect measurements of the glottic area. Therefore, in the present study the scales were placed strictly on the same plane as the vocal cords and photographed at a distance of 30 cm to provide accurate measurements.

**Figure 1.**
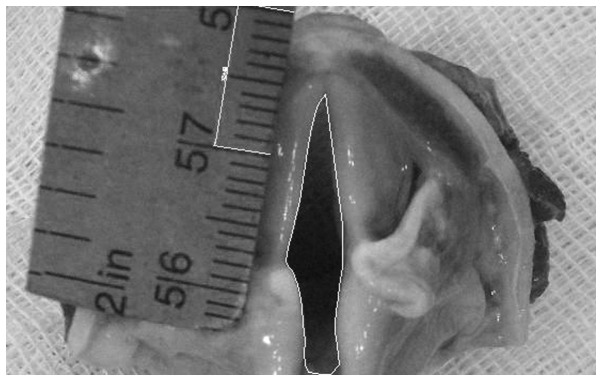
Primitive glottis of the *ex vivo* group. The white lines are polylines of the glottic area. The scale is a standard for the glottic area. Software measured the area of glottis with the scale and the polylines.

The processing of all images was completed using AutoCAD2004 image software. An image of the primitive glottis is shown in Fig. 1, with white lines which are polylines of the glottic area. The scale is a standard for the glottic area.

The experimental results of the *ex vivo* group are shown in Table I. The 3 sets of data are normally distributed. A paired t-test of the areas of the primitive glottises and glottises with closed thyroid cartilage revealed no significant differences (P>0.05). However, significant differences were revealed by a paired t-test of the areas of the primitive glottises and glottises with opened thyroid cartilage (P<0.05). The interior of the larynx in cross-section is triangular. In the experiments on the *ex vivo* group, attempts were made to confirm whether changing the shape of the glottis (with the front part resected) to a trapezoid by suturing the sternohyoid muscle to the laryngeal lumen, significantly decreases the area of the glottis. The statistical data above show that area of the glottis was enlarged in certain cases by changing its shape to a trapezoid.

**Figure 2.**
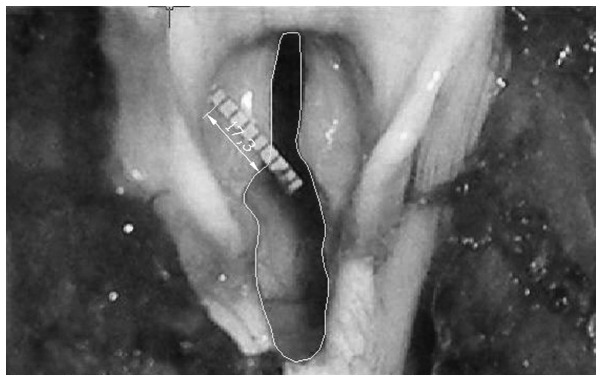
Glottis following modified frontolateral partial laryngectomy. The white lines are polylines of the glottic area. The scale is a standard for the glottic area. Software measured the area of glottis with the scale and the polylines.

A glottis following modified frontolateral partial laryngectomy is shown in Fig. 2. The white lines are polylines of the glottic area and the scale is a standard for the vocal area.

### Experimental results of the in vivo group

The experimental results of the *in vivo* group are shown in Table II. ANOVA was performed on all data and the Newman-Keuls method was used for group comparisons. No significant differences were observed between the glottic area before and after cordectomy (Q=0.047, P>0.05) or between the glottic areas prior to cordectomy and following laryngoplasty (Q=8.78, P>0.05), which demonstrates that modified frontolateral partial laryngectomy does not reduce the glottic area more than cordectomy does. However, the tissue excised by the cordectomy did not include the anterior commissure. When the anterior commissure is involved in carcinoma, the cordectomy is not a feasible therapy. No significant difference was identified between the glottic area prior to surgery and following laryngoplasty (Q=8.74, P>0.05). The statistical data revealed that the glottic area was not reduced significantly following laryngoplasty, which supports the feasibility of modified frontolateral partial laryngectomy without tracheotomy.

## Discussion

Conventionally, laryngofissure and cordectomy have been the primary means of eradicating early and selected invasive glottic squamous cell carcinomas (3). A number of studies have reported the efficacy of microendoscopic laser surgery in obtaining success rates in early cancers similar to conventional partial laryngectomy (7,8). However, when the anterior commissure is involved, partial laryngectomy is the optimal treatment option for glottic cancers since the anatomical region is not easily visualized endoscopically and safe excision margins may be compromised. Furthermore, the anterior commissure lacks an anatomical barrier to regional spread such as the adjacent perichondrium of the thyroid cartilage. Consequently, the intrinsic vulnerability of this structure may lead to unrecognized microinvasion or macroinvasion, resulting in the mistreatment of highly progressed T4a cancers that are incorrectly diagnosed as early glottic lesions (2). Therefore, partial laryngectomy is considered to be superior to laser surgery in the treatment of glottic cancer involving the anterior commissure.

Tracheotomy is routinely performed in patients undergoing partial laryngectomy due to the high risk of postoperative dyspnea originating from a narrowed laryngeal lumen or laryngeal edema. Postoperative care for the tracheotomy is burdensome for the patient and provider and is associated with prolonged hospitalization and significant morbidity. Even temporary tracheotomy is associated with increased complication rates, suggesting that prophylactic tracheotomy at the time of surgery is less than ideal. Although Muscatello *et al*(3) and Wolfensberger and Dort (9) have reportedly avoided tracheotomy in the treatment of glottic carcinomas with cordectomy via laryngofissure or endoscopic laser surgery, the feasibility of frontolateral partial laryngectomy without tracheotomy remains uncertain. In 2005, Brumund *et al*(10) reported a series of 270 patients with invasive glottic squamous cell carcinomas managed with frontolateral partial laryngectomy without tracheotomy. The present study examined the efficacy of a novel surgical approach, the modified frontolateral partial laryngectomy without tracheotomy, in achieving tumor control and restoring proper laryngeal function (11).

The feasibility and effectiveness of the modified frontolateral partial laryngectomy are functions of the anatomical association between the laryngeal lumen and its surrounding structures. The interior of the larynx in cross-section is triangular due to the contour of the thyroid cartilage. Geometrically, the area of a triangle is less than that of a trapezoid or rectangle of equivalent base and height. In the present approach, we aimed to transform the natural triangular contour into a trapezoid to achieve a greater cross-sectional area. To achieve this, the sternohyoid muscle was sutured to the laryngeal lumen and the thyroid lamina was vertically incised, resulting in abduction of the anterior part of the thyroid cartilage. In addition, the muscular fascia was retroflexed and sutured to the contra-lateral side, bringing the larynx into the desired trapezoidal or rectangular conformation. To expand the breadth of the laryngeal cavity, the sternohyoid fascia was reverted, covering the anterior larynx and completing the ladder-shaped lumen. Although the anteroposterior diameter of the neolarynx was decreased, the cross-sectional area was sufficiently enlarged to allow normal respiration, even in the absence of an endotracheal tube.

In the present study, on the basis of clinical study, animal model establishment and computer technology, it was demonstrated that expanding the anterior end of the laryngeal cavity and changing the shape from the original triangle into a trapezoid with equal bottom length and height was able to considerably increase the effective respiratory area. This result demonstrates the theoretical basis of modified frontolateral partial laryngectomy and validates its efficacy and feasibility.

In conclusion, these animal experiments demonstrated the feasibility of modified frontolateral partial laryngectomy without tracheotomy. The present data indicate that it is a safe and reliable method for excising the anterior 20% of the vocal cord and thyroid cartilage without the necessity of tracheotomy. This procedure, therefore, represents a new, less invasive technique for the treatment of glottic squamous cell carcinoma.

## Figures and Tables

**Table I.** Experimental data concerning glottis area in the *ex vivo* group.

	Primitive glottis (mm^2^)	Glottis with sutured anterior margins of thyroid cartilage (mm^2^)	Glottis with anterior ends of thyroid cartilage opened using a cartilage flap (mm^2^)
No. 1	48.41	19.52	61.15
No. 2	32.61	15.14	39.8
No. 3	54.78	27.57	54.18
No. 4	16.28	9.91	38.65
No. 5	40.37	14.67	39.37
No. 6	45.81	30.05	66.22
Mean	39.71	19.48	49.90
SD	13.71	7.89	12.25
t-score		5.74	2.47
P-value		0.023	0.057

**Table II.** Experimental data concerning glottis area in the *in vivo* group.

	Glottis before operation (mm^2^)	Glottis after laryngofissure (mm^2^)	Glottis after laryngoplasty (mm^2^)
No. 1	49.83	54.28	43.58
No. 2	17.35	13.55	13.53
No. 3	24.62	14.68	12.28
No. 4	33.79	26.64	23.28
No. 5	29.16	28.91	18.17
No. 6	45.53	51.57	37.06
No. 7	36.96	40.4	29.16
No. 8	18.33	18.54	15.16
No. 9	62.68	68.17	46.22
No. 10	65.04	67.02	57.45
Mean	38.33	38.38	29.59
SD	17.08	20.92	15.78
Q-value	-	0.05	8.74
P-value	-	0.98	0.25
